# Early marginal bone stability of dental implants placed in a transalveolarly augmented maxillary sinus: a controlled retrospective study of surface modification with calcium ions

**DOI:** 10.1186/s40729-017-0111-5

**Published:** 2017-12-04

**Authors:** Eduardo Anitua, Laura Piñas, Mohammad Hamdan Alkhraisat

**Affiliations:** 1 Private practice in oral implantology, Clínica Eduardo Anitua, Vitoria, Spain; 2 University Institute for Regenerative Medicine and Oral Implantology - UIRMI (UPV/EHU-Fundación Eduardo Anitua), Vitoria, Spain; 3 BTI Biotechnology Institute, Vitoria, Spain; 4 Universidad Europea de Madrid, Madrid, Spain; 5 Eduardo Anitua Foundation, C/Jose Maria Cagigal 19, 01007 Vitoria, Spain

**Keywords:** Calcium, Dental implant, Implant surface, Marginal bone loss, Osseointegration

## Abstract

**Background:**

Recently, components of the extracellular cellular matrix have been assessed to enhance the biological response to dental implants. This study aims to assess the effect of surface modification with calcium ions on the early marginal bone loss of dental implants placed in a transalveolarly augmented maxillary sinus.

**Methods:**

A retrospective study of transalveolar sinus floor augmentation was conducted in a single private dental clinic. The predictor variable was the surface of the dental implant. The primary outcome was the marginal bone loss. The secondary outcomes were the intraoperative complications and the dental implant failure. Descriptive analysis was performed for patients’ demographic data and implant details.

**Results:**

Fifty-one patients with a mean age of 58 ± 11 years had a mean follow-up time of 13 months. Thirty-four dental implants had a Ca^2+^-modified hydrophilic surface, and 31 had no Ca^2^ (control). The experimental group showed a statistically significant lower marginal bone loss (0.36 ± 0.42 vs 0.61 ± 0.39 mm). However, there were no statistically significant differences in the implant survival. No implant failed in the experimental group while two implants failed in the control group.

**Conclusions:**

The modification of an acid-etched surface with calcium ions seems to reduce the marginal bone remodeling around the dental implants, placed after transalveolar sinus floor elevation.

## Background

Dental implants are nowadays the treatment of choice to replace missing teeth due to their high predictability and long-term success [1]. This success is the outcome of several cellular and molecular events that take place at the implant-bone interface. Although the process of osseointegration is not fully understood, research is ongoing to enhance and accelerate this process. Moderately rough implant surface has enhanced implant osseointegration and has increased the implant secondary stability [2, 3]. Recently, elements of the extracellular cellular matrix have been introduced to bio-activate the dental implant surface [4, 5].

Calcium is one of these elements that has been studied to enhance the osseointegration process [6, 7]. Recently, Favero et al. have compared modifications of an acid-etched surface with calcium ions (UnicCa®) against a surface modified by a nanometer-scale Discrete Crystalline Deposition (DCD™) of Calcium Phosphate [8]. The patterns of sequential healing have been similar for the two surfaces, although the UnicCa® surface showed a statistically significant higher new bone formation at 2 and 4 weeks. Moreover, the osseointegration process of UnicCa® and the SLActive® surfaces has been very similar without statistically significant differences [9].

A research is needed to study if these enhancements to the dental implant surface would improve the outcome of dental implants. It has been reported that 40% of the implant failures occur during the period of osseointegration (early failures) [10]. The presence of low-density bone is a challenging situation to achieve the success of dental implants and requires specific treatment plan and surgical protocol to minimize the risk of implant failure [11, 12]. The rehabilitation of posterior maxilla with an implant-supported prosthesis could be complicated by the presence of low-density bone [13].

For that, the aim of this study is to evaluate the early survival of UnicCa® dental implants placed in transalveolarly augmented maxillary sinus. The null hypothesis of the study is that the UnicCa® surface does not enhance implant survival nor the marginal bone stability. The principal outcome has been the marginal bone stability and as secondary outcome the implant survival.

## Methods

The manuscript was written following STROBE (Strengthening the Reporting of Observational studies in Epidemiology) guidelines. All described data and treatments were obtained from a single dental clinic in Vitoria, Spain. The time period of the study was between December 2014 and April 2016. Patients’ records were retrospectively reviewed to identify patients that fulfilled the following inclusion criteria:

- Male and female patients older than 18 years old.
- Transalveolar sinus floor augmentation.
- The insertion of dental implants.

Patients/implants were excluded if not completed with all these criteria. Patients with incomplete data were also excluded. An exemption from IRB approval of the study protocol was granted by the author’s institution as it was a retrospective study, and the evaluated medical devise had already been approved for clinical use. This study was performed following the Helsinki declaration regarding the investigation with human subjects.

The principal outcome was the marginal bone loss. The experimental group was composed of the dental implants with Ca^2+^ ions (UnicCa® surface), and the control group was composed of the implants having the same surface as the UnicCa® but without the calcium ion modification (known as Optima® surface). The surface is acid-etched to generate a multi-scale roughness at the different parts of the implant (neck, valleys, and threads) in adaptation to the different biological needs: homogenous and attenuated roughness at the neck to avoid the risk of bacterial colonization, micro-roughness at the valleys to enhance the osseointegration, and micro-roughness + pores at the threads to enhance anchorage.

### Outcome assessment

Data about patients’ age and sex were collected. Cone-beam CT scans were visualized in BTI Scan III (Biotechnology Institute, Vitoria, Spain) to measure the residual bone height and the bone density at the surgical site. The sequence of bone drilling was determined according to the bone density [14].

Implant survival determined whether the implant was still physically in the mouth or lost at the time of evaluation. To assess the marginal bone stability, the distance between the uppermost point of the implant platform and the most coronal bone-implant contact was measured mesial and distal to the implant by a computer software (Sidexis, Sirona, USA). Implant length was used to calibrate the linear measurements on the radiograph.

### Surgical procedure

The plasma rich in growth factors (PRGF) was prepared using the Endoret® system following the manufacturer instructions (BTI Biotechnology Institute, Vitoria, Spain). The technique for transalveolar sinus floor elevation is explained elsewhere [15]. Briefly, conventional drills working at low speed (150 rpm) without irrigation was used to prepare the implant site. A frontal cutting drill was then introduced to prepare the last 1 mm of the implant alveolus. When a window (half of the sinus floor) was created, a well-retracted fibrin plug was introduced. The sinus floor could be opened further, if it was needed. A blunt hand instrument was introduced to push apically the fibrin membrane and to elevate the Schneiderian membrane, simultaneously. The area below the Schneiderian membrane was grafted by PRGF clot. Before implant insertion, the implant socket was irrigated with PRGF. The implants were inserted by a surgical motor at a torque value of 25 N cm. Then, the implant was completed seated with a calibrated torque wrench.

After completing the surgical and prosthetic phases, the patient was reviewed at 6 and 12 months during the observation period of the study.

### Statistical analysis

Data collection and analysis were performed by an independent examiner (other than restorative dentist and surgeon). A descriptive analysis of the implant location, length, diameter, bone grafting, and marginal bone loss was performed by considering the implant as the statistical unit of analysis. Shapiro-Wilk test was selected as normality test. Mann-Whitney test was applied to compare the follow-up time, insertion torque, and proximal bone loss between the study groups. Patients’ age, sex, and medical history were also analyzed. The bone type was compared with Fischer’s exact test and the number of implant failures by χ^2^ test.

The statistical significance level was 5% (*p* < 0.05). SPSS v15.0 for Windows statistical software package (SPSS Inc., Chicago, IL, USA) was used.

## Results and discussion

In this study, 51 patients participated with 65 dental implants. The mean age of the patients was 58 ± 11 years (range 38 to 72 years) at the time of surgery, and 28 were females.

The experimental group had 34 Ca^2+^-modified dental implants, and the control group had 31 dental implants (without surface modification with calcium ions).

**Fig. 1:**
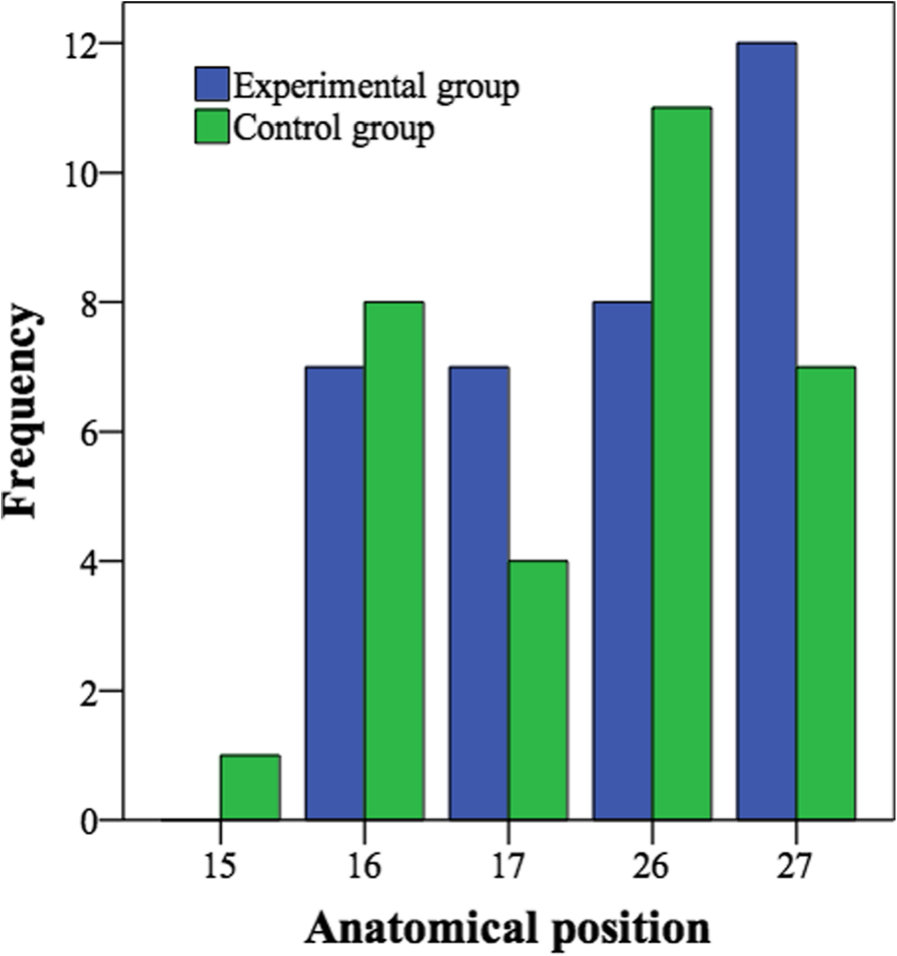
Anatomical location of the control and Ca^2+^-modified dental implants

Tables 1 and 2 show the diameters and lengths of the placed dental implants in the experimental and control groups, respectively. Figure 1 shows the anatomical position of the dental implants in the study groups. The residual alveolar bone was of type II (12 implants), type III (16 implants), and type IV (6 implants) in the experimental group. Table 3 shows the bone type in the control group that had significantly more bone of better quality. Dental implants were placed at a mean insertion torque > 30 N cm in both groups (Table 3). The healing time was 4 months. They were mainly supporting fixed screw-retained prostheses, and delayed implant loading was performed.

**Table 1:** Length and diameter of the dental implants in the experimental group

		Diameter (mm)					Total
		4.25	5.00	5.50	6.00	6.25	
Length (mm)	5.5	1	3	3	0	0	7
	6.5	0	5	12	4	2	23
	7.5	0	2	1	0	1	4
Total		1	10	16	4	3	34

**Table 2:** Length and diameter of the dental implants in the control group

		Diameter (mm)					Total
		4.25	5.00	5.50	6.00	6.25	
Length (mm)	5.5	0	1	1	0	0	2
	6.5	0	2	6	0	4	12
	7.5	0	1	10	1	2	14
	8.5	0	0	2	1	0	3
Total		0	4	19	2	6	31

**Table 3:** Outcomes of experimental and control groups

Variable		Experimental	Control	*P* < 0.05
Number of implants		34	31	
Bone type	II	35.3%	74.2%	Yes^a^
	III	47.1%	22.6%	
	IV	17.6%	3.2%	
Follow-up time (months)		13 ± 1^d^	13 ± 2^d^	No^b^
Insertion torque (N cm)		36 ± 15^d^	30 ± 15^d^	No^b^
Implant failure		0	2	No^c^
Proximal bone loss (mm)		0.36 ± 0.42^d^	0.61 ± 0.39^d^	Yes^b^

^a^Fischer’s exact test

^b^Mann-Whitney test

^c^χ^2^ test

^d^Mean ± standard deviation

No intraoperative complications were recorded. During the follow-up period (13 months), no implant failure was encountered in the experimental group. The control group had two implant failures. However, these differences were not statistically significant (Table 3). The mesial and distal bone loss in the experimental group was 0.3 ± 0.5 and 0.5 ± 7 mm, respectively. The proximal bone loss was significantly lower in the experimental group (Table 3).

The results of this study do not support the acceptance of the null hypothesis. The modification of an acid-etched surface with calcium ions (UnicCa®) has enhanced the marginal bone stability.

Maxillary sinus floor elevation using the transalveolar approach may be a valid and less invasive supplement to the lateral window technique [16, 17]. A prerequisite for using this technique is that primary implant stability could be achieved. Implant’s primary stability is the result of quantity and quality of hosting bone, the design of the implant, and the drilling technique [18]. Implant macro-design is a parameter that significantly influences implant primary stability. Ca^2+^-modified dental implants were placed following the same surgical procedure described by Anitua et al. [15] to place the same dental implant but without Ca^2+^. For that, no statistically significant differences in primary stability were found between the two dental implants.

Unlike Ca^2+^-modified dental implants, two early implant losses were observed for the same dental implants but without Ca^2+^. Moderately rough implant surface has enhanced implant osseointegration and has increased the implant secondary stability [2, 3, 19]. Hydrophilic moderately rough surfaces showed faster osseointegration compared to those with hydrophobic characteristics [20, 21]. Ca^2+^ ions have been shown to protect the hydrophilic implant surface against aging and the formation of carbon-rich species [4, 6].

Upon exposure to blood plasma, Ca^2+^-modified surface has induced surface clot formation, platelet adsorption, and activation [6]. By using a peri-implant gap model in rabbit, Ca^2+^-modified surface has significantly improved peri-implant bone volume and density at 2 weeks and bone-to-implant contact at 8 weeks [6]. Ca^2+^-modified surface presented a significantly more new bone formation at 2 and 4 weeks compared to a surface modified by nanometer-scale discrete crystalline deposition of calcium phosphate [8].

In this study, the modifications of an acid-etched surface with calcium ions have significantly decreased the marginal bone loss. Preservation of the crestal bone has been higher in Ca^2+^-modified implants compared to unmodified implants. One of the criteria of dental implant success as defined by Buser et al. [22] and modified by Albrektsson et al. [23] is the absence of persistent peri-implant bone resorption greater than 1.5 mm during the first year of loading and 0.2 mm per year during the following years. Östman et al. have documented the outcomes of dental implants modified with nanometer-scale discrete crystalline deposition of calcium phosphate [24]. The dental implants have been immediately loaded by the fixed prostheses in both maxillary and mandibular regions. The average marginal bone resorption was 0.37 ± 0.39 mm during the first year in function. This outcome might be related to the implant surface modification.

This study was limited by the retrospective design, data dependency on the accuracy of the patients’ record, and the short follow-up. Further prospective controlled studies with a long-term follow-up are required. The use of panoramic radiographs could be a source of error in measurement that was reduced by performing a 1:1 calibration of the radiograph. This would render the measurements sufficiently accurate for clinical use [25].

## Conclusions

The modification of an acid-etched surface with calcium ions (UnicCa®) seems to enhance the marginal bone stability of dental implants, placed after transalveolar sinus floor elevation.

## Abbreviations

PRGF: Plasma rich in growth factors
STROBE: Strengthening the Reporting of Observational studies in Epidemiology
